# Perceived Dangerousness as Related to Psychiatric Symptoms and Psychiatric Service Use – a Vignette Based Representative Population Survey

**DOI:** 10.1038/srep45716

**Published:** 2017-04-03

**Authors:** Julia F. Sowislo, Franca Gonet-Wirz, Stefan Borgwardt, Undine E. Lang, Christian G. Huber

**Affiliations:** 1 Universitäre Psychiatrische Kliniken (UPK), Universität Basel, Wilhelm Klein-Str. 27, CH-4002 Basel, Switzerland

## Abstract

Perceptions of dangerousness are an influential component of mental health stigma and can be driven by the display of psychiatric symptoms and the use of psychiatric service institutions. Yet, no previous study compared symptoms and service use associated perceptions of dangerousness. Therefore, we conducted a representative survey (*N* = 2,207) in the canton of Basel-Stadt, Switzerland. Participants were asked to answer the perceived dangerousness scale with respect to a vignette that either depicted psychiatric symptoms of a fictitious character or a psychiatric service institution the fictitious character had been admitted to. Between the vignettes, type of symptoms, type of psychiatric service, dangerousness, and gender were systematically varied. Perceived dangerousness was significantly lower as related to psychiatric service use than related to psychiatric symptoms. Overall, symptoms of alcohol dependency, behavior endangering others, and male gender of the fictitious character tend to increase perceived dangerousness. Furthermore, being hospitalized in a psychiatric unit at a general hospital or the rater being familiar with psychiatric services tends to decrease perceived dangerousness. Effective anti-stigma initiatives should integrate education about dangerousness as well as methods to increase familiarity with psychiatry. Additionally, an integration of modern psychiatry in somato-medical care institutions might decrease stigmatization.

Individuals with mental illness are exposed to extreme stigmatization and devaluation^1^. The negative consequences of stigmatization, such as increased anxiety and stress, decreased functional outcome, and lower quality of life can be considered as a ‘second disease’^2^,^3^. Accordingly, a wealth of scientific literature has been published on mental health stigma, some of which is inconsistent in its findings. These inconsistencies might be partly due to the fact that stigma consist of different facets. The most commonly measured facets of stigma are (increased) desire for social distance and perception of (higher) dangerousness of the target group^4^. Individuals with mental illness are commonly perceived as being relatively dangerous, as multiple representative studies have shown^4^. This finding is mirrored in everyday life, for instance by stereotyped media portrays that highlight a high risk for violence in mental illness^3^. Yet, these perceptions are biased: Although there is a significantly elevated risk for violence, the risk is small and the majority of individuals with mental illness are not violent^5^. Perceptions of dangerousness gain special importance as they are hypothesized to influence other facets of stigmatization such as desire for social distance^6^ or for coercion^7^.

Perceptions of dangerousness might be influenced by different sources of information. One source might be information about symptoms or deviant behavior (dangerousness associated with psychiatric symptoms). However, psychiatric symptoms are, in contrast to other stigmatized conditions such as ethnicity, often invisible. Consequently, information about psychiatric service use (e.g., the information that a person has been treated in a psychiatric hospital) is another important source of perceptions of dangerousness (dangerousness associated with psychiatric service use). This in turn negatively influences psychiatric service use and adherence: To avoid stigmatization, individuals with mental illness tend to avoid the treatment they are in need for^8^,^9^.

Research on perceived dangerousness associated with psychiatric symptoms has revealed differences across different bundles of symptoms/psychiatric diagnoses. More precisely, individuals with alcoholism are perceived as more dangerous than individuals with psychosis^10^. Yet, research on the stigmatization of individuals with symptoms of personality disorders is still scarce. The only existent studies suggest that mental health professionals^11^,^12^ perceive individuals with borderline personality disorder (BPD) as more dangerous than individuals with alcoholism. However this effect might be specific to professionals, as individuals with BPD tend to evoke a intense disorder-specific countertransference in treatment^13^. Thus, supplemental studies in the general population are needed.

To the best of our knowledge, there is as yet no research on dangerousness associated with psychiatric service use that studies differences across different types of service institutions. However, there is one previous study showing that hospitalizations in a psychiatric unit at a general hospital evoke less desire for social distance (the latter being another facet of stigmatization as mentioned above)^14^. It is plausible that the concrete type of institution also influences perceptions of dangerousness. For instance, by often being geographically dislocated, psychiatric hospitals might evoke associations of confinement and thus foster perceptions of dangerousness^15^.

In addition, familiarity with psychiatric illness^16^ is known to influence the stigma associated with displaying psychiatric symptoms. For instance, Angermeyer *et al*.^17^ showed that individuals who are familiar with psychiatric illness are less likely to believe that people with psychiatric illnesses are dangerous.

The present study is the first to compare the amount of perceived dangerousness associated with psychiatric symptoms versus with psychiatric service use. Furthermore, we pitted perceived dangerousness associated with symptoms of BPD in the general population against perceived dangerousness associated with symptoms of alcohol dependency and schizophrenia. Finally, our study is the first to compare the amount of perceived dangerousness across different psychiatric inpatient institutions, including forensic-psychiatric settings.

## Methods

### Sample and Procedure

Data come from a survey on psychiatric service use and stigmatization that was conducted from autumn 2013 to spring 2014 among citizens of Basel, Switzerland^14^. This study was approved by the local ethics committee (“Ethikkommission Nordwest- und Zentralschweiz”, EKNZ 2014-394) and conducted according to the Declaration of Helsinki. The current study used questionnaires that were sent out to the general population aged 18 or above. It included a letter detailing study procedures, stating that participation was completely optional, and that non-participation would not be followed by any consequence. Persons agreed to participate by sending in their questionnaire. This procedure was accepted as informed consent by the EKNZ. The manuscript does not contain information or images that could lead to identification of a study participant.

A sample of 10,000 individuals was randomly drawn from the cantonal resident register and was mailed study material. To be eligible, participants had to be registered in a private household in Basel-City, Bettingen, or Riehen for a minimum of two years, had to be aged from 18 to 65 years, and had to have sufficient knowledge of the German language. This approach was chosen in a consensus procedure together with the Statistical Office of Basel-City and an external advisory committee to generate a representative study sample.

Participants had to read a vignette and answer several questionnaires. Vignettes described a fictitious character and depicted either the psychiatric symptoms of the character (case vignette) or a clinic the character had been admitted to (clinic vignette). Between both types of vignettes, gender and dangerousness of the fictitious patient were systematically varied: Half of the case vignettes contained a male (Sebastian M.) versus a female character (Katrin M.). Moreover, it was explicitly stated that within the last month the main character (case vignette) or the patients of the clinic (clinic vignette), either displayed no dangerous behavior, self-endangering behavior, or behavior endangering others. Additionally, between the case vignettes, type of psychiatric symptoms was systematically varied. They either described a case of acute psychotic disorder, a case of alcohol dependency, or a case of borderline personality disorder, not being labeled directly, but with symptoms fulfilling the relevant DSM-V criteria^18^. Case vignettes were constructed based on the vignettes that had been used in previous stigma research^19^,^20^. However, some modifications were introduced to eliminate potential confounders. Apart from the characteristics that were systematically varied (i.e., symptoms, gender, and dangerousness), all other information (e.g., age of the fictitious character) was kept constant between the case vignettes. Before being used in the main survey, vignettes were submitted to *N* = 18 psychiatrists and clinical psychologists for blind diagnostic allocation. Supporting the validity of the case vignettes, each diagnosis was labeled correctly by all of the clinical experts.

Moreover, between the clinic vignettes, type of the psychiatric service institution to which the fictitious character was admitted was systematically varied. Vignettes either described a general hospital including a psychiatric unit, or a psychiatric hospital, or a psychiatric hospital also including a forensic unit. Thus, we systematically manipulated the characteristics that are per definition specific to the respective types of clinics, as well as gender and dangerousness, whereas all other information (e.g., carrying capacity of the fictitious clinic) was kept constant across the clinic vignettes.

Considering the variations described above there were 36 individual vignette conditions, so that each condition was sent to about 277 participants (for the concrete wording of exemplary vignette conditions see Sowislo *et al*.^14^).

The final sample consisted of 2,207 individuals (61.5% female, 66.5% Swiss citizens, 44.7% single), reflecting a response rate of 22.1%. Mean age of participants was 43.4 years (*SD* = 13.4). 6.2% percent had completed the obligatory 9 school years, 51.3% had completed secondary education (approximately 12 years), and 42.0% had a university degree. There were no significant differences in number of respondents per individual vignette condition, nor for the case (χ^2^ (17, *N* = 1107) = 19.00, *p* = 0.329) neither for the clinic vignettes (χ^2^ (17, *N* = 1100) = 6.84, *p* = 0.986).

To assess representativeness of our sample, respondent characteristics were compared to official census data as published in the statistical almanac of Basel-City^21^. However, this comparison has to be interpreted with caution, as the data available from the statistical almanac represent the whole population of Basel-City without the restrictions posed by our in- and exclusion criteria. At the end of 2013, 191,606 persons were registered in Basel-City. 52.0% were of female gender, 67.0% were Swiss citizens, and 45.7% were single. Mean age was 42.9 years. 17.5% had completed obligatory school, 48.6 secondary education, and 32.5% higher/university education. The comparison shows that questionnaires were sent out to over 5.2% of the population. The study sample represents more than 1.2% of the total population and can be assumed to be representative with regard to age, nationality, martial status, and living situation. However, there seems to be an overrepresentation of women and of persons with higher education in our sample.

### Measures

#### Perceived Dangerousness

Perceived dangerousness was measured with the dangerousness scale^22^,^23^. The scale consists of eight items that assess individual beliefs about the dangerousness of the fictitious person in the vignette. Responses were made on a 4-point scale and a composite (with higher values indicating higher perceived dangerousness) was derived by totaling the sum of all items.

#### Familiarity with Mental Illness

Familiarity with mental illness was measured with four items asking participants whether they themselves had ever undergone psychiatric inpatient or outpatient treatment, and whether anyone within their family or anyone within their circle of friends had ever undergone psychiatric treatment. Similar to Angermeyer *et al*.^17^, we used this information to construct four hierarchical categories representing the degree of familiarity with mental illness: (1) the participant herself/himself, (2) a family member of the participant, (3) or a friend of the participant has undergone psychiatric treatment, or (4) none of these possibilities applies to the participant. If more than one category applied for one respondent, we chose the one with the highest familiarity.

### Statistical Analysis

All statistical analyses were conducted using the SPSS 19 statistical package for Windows (IBM Corporation, Armonk, NY, USA). We examined differences in social distance between case and clinic vignettes using an independent *t*-test. Then, we conducted two separate multiple regression analyses for the case and clinic vignettes respectively, with perceived dangerousness as dependent variable. Categorical predictors with more than two categories (i.e., type of psychiatric symptoms, type of psychiatric service, degree of familiarity with psychiatric illness, and dangerousness) were entered as dummy variables. In the first regression analysis, type of psychiatric symptoms, gender and dangerousness of the fictitious person in the vignette, and familiarity with psychiatric illness of the respondent were entered as independent variables. In the second regression analysis, type of psychiatric service, gender of the fictitious person in the vignette, dangerousness, and familiarity with psychiatric illness of the respondent were entered as independent variables. Multiple regression analysis offers a significance test for the difference between the chosen reference category (e.g., alcohol dependency) and each of the chosen comparison groups (e.g., psychosis and BPD), respectively. However, it does not allow statistically comparing the comparison groups (e.g., psychosis versus BPD) in a direct fashion. To do so, we statistically pitted the available standardized regression weights (e.g., alcohol dependency versus acute psychotic disorder and alcohol dependency versus BPD) against each other, using a *t*-test for the difference between regression weights from the same sample (overlapping coefficients). As suggested by Cohen *et al*.^24^ the standard error of the difference between such two regression weights (i.e., β_i_ and β_j_) was of the form:


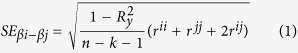


In this equation, *i* and *j* denote the two (dummy) variables, *n* is the number of participants, *k* is the number of independent variables, *R*^2^ is the coefficient of determination, and r^ii^, r^jj^, and r^ij^ represent corresponding elements of the inverted correlation matrix.

## Results

First, an independent *t*-test showed that perceived dangerousness was significantly higher (*t* = 11.33, *p* < 0.001) with respect to the case vignettes (*M *=* *10.38, *SD *=* *4.29) than with respect to the clinic vignettes (*M *=* *8.16, *SD* = 4.87).

Second, the regression predicting dangerousness of the fictitious person in the case vignettes revealed that familiarity, type of psychiatric symptoms, dangerousness, and gender of the fictitious person were significant predictors (see Table 1). Concerning familiarity, perceived dangerousness was significantly lower when the participant, a family member, or a friend versus none of them had undergone psychiatric treatment. Furthermore, *t*-tests comparing the standardized regression weights showed that there was no significant difference in perceived dangerousness between a family member versus a friend having undergone psychiatric treatment (*t* = −1.15, *p* = 0.252) and the patient herself/himself versus a family member having undergone psychiatric treatment (*t* = −0.34, *p* = 0.737). Concerning type of psychiatric symptoms, symptoms of alcohol dependency tended to elicit significantly more perceived dangerousness than symptoms of BPD, whereas there was no corresponding difference between symptoms of alcohol dependency and symptoms of psychotic disorder. Again, a *t-*test comparing the standardized regression weights indicated that perceived dangerousness does not significantly differ between BPD and acute psychotic disorder (*t* = −0.37, *p* = 0.712). Concerning dangerous behavior, information that the fictitious person endangered others elicited significantly more perceived dangerousness than information that the fictitious person endangered herself/himself or endangered no one at all. Furthermore, at a trend level, information that an individual endangered herself/himself provoked more perceived dangerousness than information that an individual endangered no one at all (*t* = −1.65, *p* = 0.099). Finally and replicating prior research, the description of a female fictitious person provoked significantly less perception of danger than the description of a male fictitious person.

Third, the regression predicting dangerousness of the fictitious person in the clinic vignettes revealed that familiarity, type of psychiatric symptoms, dangerous behavior, and gender of the fictitious person were significant predictors (see Table 2). Concerning familiarity, the pattern of the first regression analysis was replicated. More precisely, the fact that the participant herself/himself, or a family member, or a friend had been in psychiatric treatment significantly decreased perceived dangerousness. However, there were no significant differences in perceived dangerousness between different degrees of familiarity (patient herself/himself versus family member: *t* = −0.74, *p* = 0.459; family member versus friend: *t* = −0.92, *p* = 0.358). Concerning type of psychiatric service, patients of a general hospital with a psychiatric unit tended to elicit less perception of dangerousness than patients of a psychiatric hospital with a forensic unit. Patients of a psychiatric hospital without a forensic unit provoked similar perceived dangerousness as patients of a psychiatric hospital with a forensic unit. A *t-*test comparing the standardized regression weights further revealed no difference in perceived dangerousness between patients of a general hospital with a psychiatric unit and patients of a psychiatric hospital without a forensic unit (*t* = 0.29, *p* = 0.771). Concerning dangerous behavior manipulated across the vignettes, information that an individual was inpatient at a hospital some of whose patients endanger others elicited significantly more perceived dangerousness than information that none of the other patients had shown dangerous behavior. However, and in contrast to the previous analyses, the degree of dangerousness did not differ for some of the other patients showing behavior endangering others versus self-endangering behavior. Furthermore, information that some of the patients endanger themselves does not provoke significantly more perceived dangerousness than information that the patients endanger no one at all (*t* = −1.07, *p* = 0.285). Last and as in the previous analysis, male fictitious persons yielded higher ratings of perceived dangerousness than female fictitious persons.

## Discussion

### Main Findings

This vignette and questionnaire based study is the first to show that individuals with mental illness are believed to be more dangerous than individuals using psychiatric services. Dangerousness associated with psychiatric symptoms was diagnosis-specific: While symptoms of alcohol dependency elicited stronger perception of dangerousness, symptoms of BPD and acute psychotic disorder did so to a lesser extent. Dangerousness associated with psychiatric services was partly institution-specific: Individuals were perceived more dangerous when they used a psychiatric hospital with a forensic unit in contrast to a general hospital which included a psychiatric unit. However, this difference disappeared when the psychiatric hospital did not include a forensic unit. Furthermore, concerning the relative amount of dangerousness, it made no significant difference whether the psychiatric hospital included a forensic unit or not. The respondent being familiar with psychiatric illness decreased both symptom and service-use related stigma. Behavior endangering others and self-endangering behavior both increased perceived dangerousness associated with psychiatric symptoms (e.g., in case the person carrying symptoms was displaying this behavior) as well as dangerousness associated with psychiatric service use (e.g., in case the persons used a service institution some of whose inpatients were displaying this behavior). Finally, male persons tended to elicit more perceived dangerousness than female persons.

### Variables Connected with Perceived Dangerousness

Our findings demonstrate that individuals displaying psychiatric symptoms (case vignette) are believed to be more dangerous than individuals using psychiatric institutions (clinic vignette). This is in line with previous findings that demonstrated a similar pattern of results for a familiar component of stigma, namely desire for social distance^14^. Together these findings highlight that a distinction along the source of stigma (symptoms vs. service use) might be useful for different components of stigma. As stigma emerging from symptoms versus service use seems to be relatively independent (our study systematically varies one source of stigma while simultaneously holding the other constant) anti-stigma campaigns should target both.

With regard to type of symptoms, alcohol dependency was believed to be the most dangerous condition. This might be at least partly due to differences in the frequency of personal experience^25^. More people in the general population might have been confronted with alcohol-related violence than with violence related to the two other conditions, because alcohol consumption is relatively prevalent in non-clinical as well as clinical settings^26^. Yet, this perceived rang-order might accurately mirror the corresponding rank-order in risk estimates for violence. More precisely, in their meta-analysis Fazel *et al*.^27^ showed that the risk of violence in individuals with substance abuse is higher than in individuals with psychotic disorder. Thus, although the amount of dangerousness of individuals with psychiatric symptoms might be overestimated, differences in dangerousness between different symptom conditions might be judged accurately. This hypothesis should be investigated in future studies in the general population.

Our study is the first to show that perceived dangerousness is directly connected to the type of psychiatric service used by an individual, independent of the psychiatric diagnosis or symptoms displayed. Indeed, in our study we observed that, although diagnoses treated did not vary, the use of a psychiatric hospital is eliciting more dangerousness than the use of a general hospital that includes a psychiatric unit. This is interesting as – in contrast to psychiatric symptoms – the structure of psychiatric services provided in an area could be changed. As the stigma associated with psychiatric treatment tends to be institution-specific, an integration of modern psychiatry in somato-medical care institutions might be a possibility to decrease stigmatization and increase the utilization and thereby improve the outcome of psychiatric treatment. Moreover, concerning the degree of stigmatization, in our study it makes no difference whether the psychiatric hospital includes a forensic unit or not. This observation supports the view that – despite being informed adequately about the patient population– the general population is not able to distinguish a common psychiatric hospital where medical care is provided from a forensic hospital, where legally detained criminals are treated.

Finally, our study shows that familiarity with mental illness decreases perceptions of dangerousness. In the context of other studies, a difference between having had contact to a person in psychiatric treatment or not seems to replicate across studies^17^. Clearly, to build non-biased, realistic, and available representations of dangerousness, the general public should have multiple opportunities to contact and interact with individuals with psychiatric illness.

### Strengths and Limitations

Our study was the first of its kind to systematically investigate differences in stigma between inpatient settings which might be especially informative to concrete service institutions and general practitioners. However, the findings should be viewed in the context of the study’s limitations. A first limitation consists in possible threats to external validity. The response rate of 22.1% might account for selection and non-response biases (e.g., leading to increased participation of women and of persons with higher education). Participation of persons with a relatively high level of education may additionally have been facilitated due to the questionnaire-based method. Moreover, participation was limited to inhabitants of the canton of Basel-Stadt, an urban environment, which might also be reflected in differences in stigmatization of psychiatry across culturally and politically distinct areas. A second limitation pertains to the internal validity of our interpretation regarding familiarity with psychiatric illness. As we used cross-sectional data, a causal interpretation of the association between familiarity and perceptions of dangerousness cannot be ensured. It is also possible that individuals with higher perceptions of dangerousness avoid psychiatric treatment and/or contact to individuals who sought psychiatric treatment. Although there is one study^28^ that suggests that familiarity has a causal role for perceived dangerousness, future longitudinal studies should complete the picture. However, due to the experimental manipulation of symptoms, service institutions, gender, and dangerous behavior, our conclusions referring to these constructs are highly internally valid.

## Conclusion

Although the majority of individuals with a mental illness is not violent, our results suggest that this finding is not reflected in attitudes of the general population. Individuals displaying psychiatric symptoms or using psychiatric service institutions are perceived to be dangerous. Therefore, effective anti-stigma initiatives should integrate education about dangerousness as well as methods to increase familiarity with psychiatry. Finally, an integration of modern psychiatry in somato-medical care institutions and health centers might decrease perceived dangerousness. A corresponding increase in utilization of psychiatric treatment and in well-being of our patients should be further explored in clinical practice and research.

## Additional Information

**How to cite this article:** Sowislo, J. F. *et al*. Perceived Dangerousness as Related to Psychiatric Symptoms and Psychiatric Service Use – a Vignette Based Representative Population Survey. *Sci. Rep.* **7**, 45716; doi: 10.1038/srep45716 (2017).

**Publisher's note:** Springer Nature remains neutral with regard to jurisdictional claims in published maps and institutional affiliations.

## Figures and Tables

**Table 1:** Linear Model of Predictors of Change in Perceived Dangerousness in the Case Vignettes.

	*B*	β	*SE B*	*p*
Constant	15.52 (14.46, 16.69)		0.56	*p* < 0.001
Familiarity				
Friends vs. none	−1.48 (−2.30, −0.71)	−0.15	0.44	*p* = 0.001
Family vs. none	−2.22 (−3.01, −1.47)	−0.24	0.42	*p* < 0.001
Self vs. none	−2.48 (−3.23, −1.72)	−0.27	0.42	*p* < 0.001
Diagnosis				
Borderline vs. alcohol dependency	−0.62 (−1.23, −0.03)	−0.07	0.30	*p* = 0.038
Psychosis vs. alcohol dependency	−0.43 (−1.02, −0.17)	−0.05	0.30	*p* = 0.154
Dangerousness				
None vs. endangering others	−2.53 (−3.12, −1.86)	−0.28	0.30	*p* < 0.001
Self-endangering vs. endangering others	−1.60 (−2.19, −1.03)	−0.18	0.30	*p* < 0.001
Gender				
Female vs. male	−0.98 (−1.47, −0.47)	−0.11	0.25	*p* < 0.001

*Note. R*^*2*^ = 0.114 (*p* < 0.001); 95% bias corrected and accelerated confidence intervals (CI) are reported in parentheses. Confidence intervals and standard errors (SE) are based on 1000 bootstrap samples. *B:* unstandardized regression weight; *β:* standardized regression weight.

**Table 2:** Linear Model of Predictors of Change in Perceived Dangerousness in the Clinic Vignettes.

	*B*	β	*SE B*	*p*
Constant	13.58 (12.05, 15.07)		0.72	*p* < 0.001
Familiarity				
Friends vs. none	−2.67 (−3.80, −1.56)	−0.23	0.58	*p* < 0.001
Family vs. none	−3.32 (−4.43, −2.22)	−0.32	0.55	*p* < 0.001
Self vs. none	−4.03 (−5.16, −1.97)	−0.40	0.55	*p* < 0.001
Clinic				
Psychiatry vs. forensic	−0.46 (−1.16, 0.25)	−0.04	0.36	*p* = 0.200
General hospital vs. forensic	−0.82 (−1.51, −0.14)	−0.08	0.36	*p* = 0.022
Dangerousness				
None vs. endangering others	−1.29 (−1.96, −0.60)	−0.13	0.36	*p* < 0.001
Self-endangering vs. endangering others	−0.61 (−1.36, −0.07)	−0.06	0.36	*p* = 0.086
Gender				
Female vs. male	−0.81 (−1.38, −0.26)	−0.08	0.29	*p* = 0.005

*Note. R*^*2*^ = 0.071 (*p *<* *0.001); 95% bias corrected and accelerated confidence intervals (CI) are reported in parentheses. Confidence intervals and standard errors (SE) are based on 1000 bootstrap samples. ‘Psychiatry’ abbreviates a psychiatric hospital without forensic unit; ‘forensic’ abbreviates a psychiatric hospital with forensic unit. *B:* unstandardized regression weight; *β:* standardized regression weight.
